# Sexual conflict maintains variation at an insecticide resistance locus

**DOI:** 10.1186/s12915-015-0143-3

**Published:** 2015-06-29

**Authors:** Wayne G Rostant, Caroline Kay, Nina Wedell, David J Hosken

**Affiliations:** Centre for Ecology & Conservation, University of Exeter, Cornwall Campus, Tremough, Penryn, TR10 9FE Cornwall UK; Department of Biology & Bichemistry, University of Bath, Bath, BA2 7AY UK; Present address: School of Biological Sciences, University of East Anglia, Norwich, NR4 7TJ Norfolk UK

## Abstract

**Background:**

The maintenance of genetic variation through sexually antagonistic selection is controversial, partly because specific sexually-antagonistic alleles have not been identified. The *Drosophila* DDT resistance allele (DDT-R) is an exception. This allele increases female fitness, but simultaneously decreases male fitness, and it has been suggested that this sexual antagonism could explain why polymorphism was maintained at the locus prior to DDT use. We tested this possibility using a genetic model and then used evolving fly populations to test model predictions.

**Results:**

Theory predicted that sexual antagonism is able to maintain genetic variation at this locus, hence explaining why DDT-R did not fix prior to DDT use despite increasing female fitness, and experimentally evolving fly populations verified theoretical predictions.

**Conclusions:**

This demonstrates that sexually antagonistic selection can maintain genetic variation and explains the DDT-R frequencies observed in nature.

**Electronic supplementary material:**

The online version of this article (doi:10.1186/s12915-015-0143-3) contains supplementary material, which is available to authorized users.

## Background

Males and females share many traits and these are controlled by a common genetic programme [1]. However, the sexes are frequently subjected to sex-specific selection for shared traits [2–4] and this can generate intralocus sexual conflict [5–8]. This conflict is pervasive [9–12], occurring whenever males and females differ in their optimal values for shared traits [5]. Human hip-width is a putative example of sexual conflict over trait values, with wider hips favoured in females to accommodate child birth, but disfavoured in males because of mobility costs [13]. Intralocus sexual conflict has been promoted as a mechanism for maintaining genetic variation [5, 7, 14], although this is controversial as the theoretical conditions required are restrictive [15–19]. Additionally, there has been no empirical test of this possibility for naturally occurring alleles [14] because definitive sexually-antagonistic allelic variation has never been precisely identified. In fact it has been argued that it will be very difficult to map sexually antagonistic traits to single genes [18, 20, 21].

An emerging exception to this generalisation is the *Drosophila melanogaster* DDT resistance allele (DDT-R) of the gene *Cyp6g1* [22], for which we recently documented sexually antagonistic selection on a *Canton-S* genetic background [23]. DDT-R increases female fecundity and survival of their offspring [24], even in the absence of DDT (Additional file 1: Figure S2 and [24]), but decreases male fitness [23]. The finding of sexual antagonism in this background is important because *Canton-S* was isolated prior to the widespread use of DDT [25] and near fixation of DDT-R [26]. As a result, there cannot have been DDT-R/*Canton-S*-background coevolution to ameliorate the male costs DDT-R generates [27]. Put another way, selection should favour modifiers that reduce the negative fitness effects of DDT-R on males, but the *Canton-S* background should largely retain the primordial condition as there has been no coevolution between it and DDT-R. Importantly, this sexual antagonism potentially resolves a troubling paradox - while the resistance allele was present before the use of DDT, it was not until after DDT use that the allele increased in frequency [26], despite large fitness benefits for female carriers [24]. The intralocus conflict we previously documented [23] provides one potential explanation for this pattern: in the absence of pesticide, DDT-R increases female fitness, but simultaneously decreases male fitness. Thus, the sexual antagonism hypothesis could explain why variation was maintained at the DDT-R locus and why DDT-R did not fix despite its female benefits, but this remains to be tested.

Here we present a population genetic model of *D. melanogaster* DDT-R that incorporates the sex-specific fitness effects previously documented in the *Canton-S* background [22], and used this theory to examine the maintenance of genetic variation over parameter values derived from previous empirical work [23, 24]. Previous models of this nature have not been based on directly quantified measures of male and female fitness at antagonistic loci [15–19], unlike here. Our model assumes that DDT-R increases egg and larval survival through a dominant maternal effect, for which there is evidence [24]. This means that the standard diploid population genetics models of sexual antagonism must be modified. Also, we assume a direct dominant effect of the DDT-R allele on increasing the pupal survival of both sexes, of reducing the mating success of males, and of increasing the fecundity of females. This assumption is based on the evidence for dominance in DDT resistance [22, 28, 29] and various pleiotropic fitness measures [24].

Theoretical predictions were then tested in replicate experimentally evolving fly populations to empirically address whether sexual conflict can maintain genetic variation at a known, naturally occurring, sexually antagonistic allele (DDT-R), and whether this might explain the evolutionary history of this allele.

## Results and discussion

By inserting fitness estimates [23, 24] into a non-linear recursion model we generated predictions of allele frequency dynamics over time. The model terms with default parameter values are outlined in Table 1. The model yields at least two solutions (the boundary equilibria, where DDT-R is absent or fixed) and, under certain fitness parameter values a third, internal equilibrium (intermediate DDT-R frequency). It can be shown that for a stable internal equilibrium to exist the following inequalities must be true:

**Table 1:** Model terms

Term and default Canton-S parameter values	Definition
*x*_*R*_	DDT-R (i.e. *Accord* LTR*-*inserted) allele frequency
*m* = 0.28	Relative competitive mating success of DDT-R males compared to susceptible males
*f* = 2.13	Relative fecundity of DDT-R females compared to susceptible females
*e* = 1.57	Viability advantage of eggs laid by DDT-R females (*RR* and *RS*) compared to susceptible (*SS*) females
*l* = 1.13	Viability advantage of larvae of DDT-R females (*RR* and *RS*) compared to susceptible (*SS*) females
*F* = *f* × *e* × *l =* 3.79	Combined fitness advantage conferred to resistant females
*P* = 1.12	Pupal viability advantage of DDT-R flies (*RR* and *RS*) compared to susceptible (*SS*) flies
*y*_*RR*_ , *y*_*RS*_ , *y*_*SS*_	Probability that a mating male has a particular DDT-R genotype: see equations (3)
*x*_*RR*_ , *x*_*RS*_ , *x*_*SS*_	DDT-R genotype frequencies: see equations (6)
*D* = 5	DDT Resistance ratio of DDT-R (*RR* and *RS*) to susceptible (*SS*) flies (mortality of susceptible flies/mortality of DDT-R allele carrying flies).

Parameter estimates for *f*, *e*, *l* and *P* from McCart [24] and *m* from Smith *et al.* [23]

1 $$ P>\frac{2}{m+F} $$

2 $$ P<\frac{m+F}{2mF} $$

where *P* = pupal survival, *m* = the male fitness disadvantage of DDT-R and *F* = the female fitness advantage.

**Fig. 1:**
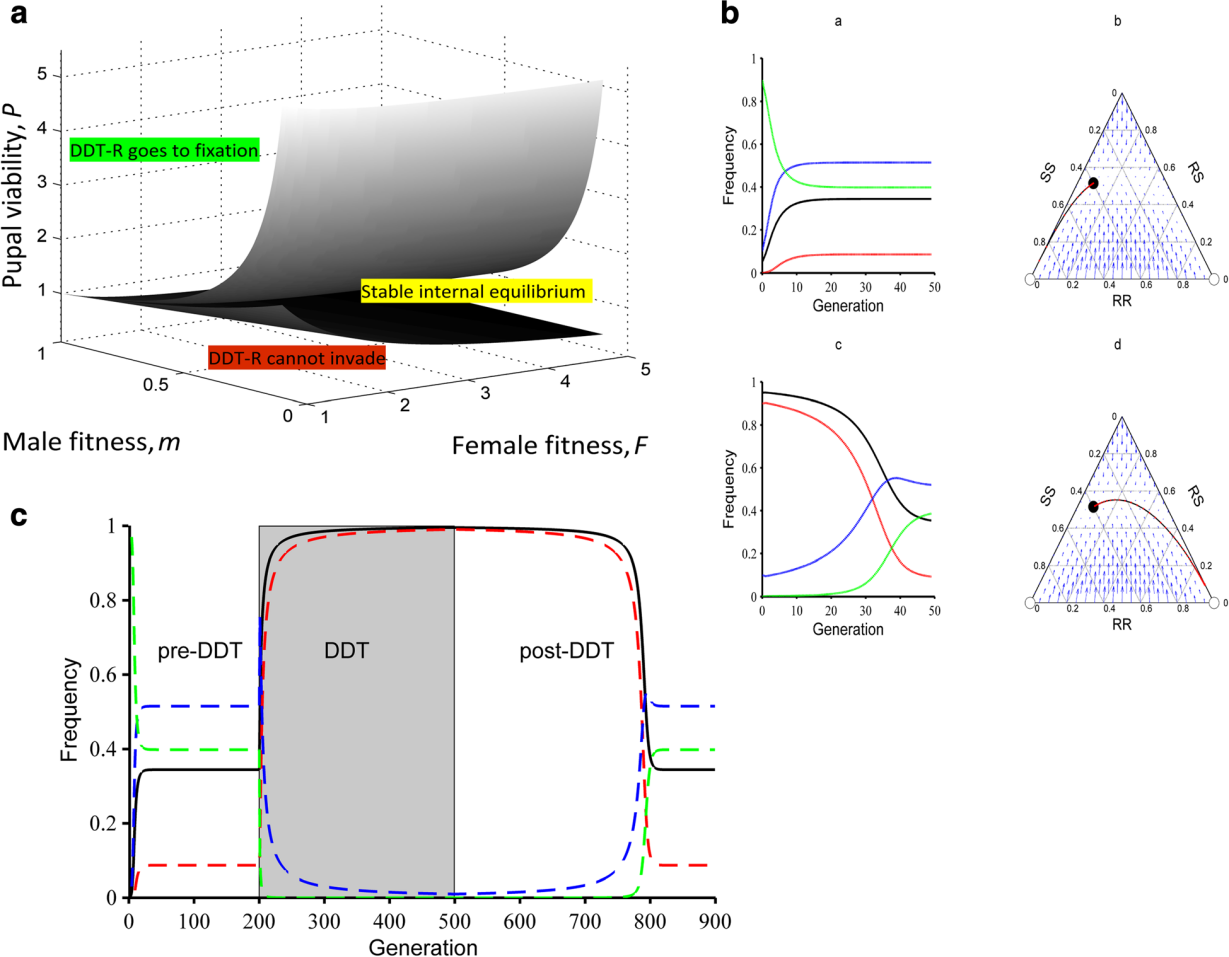
The theoretical allele frequencies with and without DDT imposed selection. **(a)** The model parameter space showing three different equilibrium regions. If the upper surface is exceeded DDT-R goes to fixation. Below the lower surface DDT-R cannot invade. A stable internal equilibrium, where both resistant and susceptible genotypes co-occur, exists in the envelope between the two surfaces. **(b)** Model DDT-R genotype and allele trajectories approach a stable internal equilibrium. Model run over 50 generations with fitness parameters at default *Canton-S* values (Table 1). (a) and (b): initial genotype frequencies *x*_*RR*_ = 0, *x*_*RS*_ = 0.1, *x*_*SS*_ = 0.9; (c) and (d) initial genotype frequencies *x*_*RR*_ = 0.9, *x*_*RS*_ = 0.1, *x*_*SS*_ = 0. In plots (a) and (c) the red line represents the frequency of *x*_*RR*_, the blue line *x*_*RS*_, the green lines *x*_*SS*_, and the black line is DDT-R. Ternary plots (b) and (d) show genotype trajectory (red dots connected by black lines), equilibria (open circles are unstable equilibria, black circle is stable equilibrium) and genotype vector field (blue arrows). **(c)** The effect of added DDT viability selection on model DDT-R genotype and allele trajectories. Fitness parameters set to default *Canton-S* values (Table 1) starting from initial genotype frequencies *x*_*RR*_ = 0, *x*_*RS*_ = 0.001, *x*_*SS*_ = 0.999. The red line is the frequency of *x*_*RR*_ , the blue line is *x*_*RS*_ , the green line is *x*_*SS*_ , and the black line is DDT-R. The internal equilibrium of 34 % in the absence of DDT selection is achieved within the first 20 generations (in the ‘pre-DDT’ period). DDT selection (shaded area) starts at generation 201 and ends at generation 500, by which time DDT-R has acquired a frequency greater than 99 %. More than 300 subsequent generations are required ‘post- DDT’ for the stable internal equilibrium to be regained

The stability of each boundary equilibrium also depends on these inequalities (Fig. 1a).

If inequality (1) is reversed, then the lower boundary equilibrium is stable and DDT-R cannot invade a susceptible population. Correspondingly, if inequality (2) is reversed, then the upper boundary equilibrium is stable and DDT-R at any initial frequency will go to fixation (Fig. 1a).

If inequalities (1) and (2) are true, explicit solutions for all internal equilibria can be found (Additional file 1). It can then be shown that the stable internal equilibrium occurs at genotype frequencies ${\widehat{x}}_{RR}=0.09,\kern0.5em {\widehat{x}}_{RS}=0.51,\kern0.5em {\widehat{x}}_{SS}=0.40$, where subscripts refer to resistance (*R*) and susceptible (*S*). This is globally stable, so regardless of the starting frequency (as long as it is neither 0 nor 1), the DDT-R allele frequency will go to a stable equilibrium of 34 % in the absence of DDT (Fig. 1b). An initially high frequency mirrors the current situation in the wild where DDT-R has reached near fixation in many global populations [26].

It takes considerably longer to reach equilibrium when starting from high DDT-R frequency when compared to an initially low DDT-R frequency (Fig. 1b) – this demonstrates that it is far easier for the resistance allele to invade a susceptible population than for the susceptible allele to invade a resistant population. The asymmetry is a direct result of the frequency-dependent selection acting on males. Thus, in a population with very low background DDT-R frequency, a resistant male does much worse than he would in a population with very high background DDT-R frequency because of the higher number of encounters with competitively superior SS males.

When simulating selection from DDT, the added viability advantage to DDT-R [24] rapidly pushes the allele towards fixation (Fig. 1c). As long as complete fixation is not achieved, removal of pesticide selection allows a return to the internal equilibrium, but at a very slow rate – it takes more than 300 generations for this to occur. If we assume (to conservatively account for variance in parameter estimates) weaker sexually antagonistic selection on the allele, DDT-R is still kept at an allelic frequency of about 0.01 in the absence of DDT, until DDT drives it to near fixation (Additional file 1: Figure S1).

By using empirically derived estimates of male and female fitness to quantify the magnitude of intralocus sexual conflict at this locus [23, 24], we find that polymorphism in resistance is maintained over a wide range of values, which contrasts with previous models [15–18] where genetic variation was maintained only in limited parameter range. This difference is attributable to previous models utilising very small selection coefficients, whereas here, we have documented sexually antagonistic selection at the allele and found it to be orders of magnitude stronger than previously assumed. That is, relaxation of the assumption of weak selection found in previous generalised models of intralocus sexual conflict make the conditions for stable polymorphism less restrictive.

To test model predictions, we established replicate, experimental fly populations with different initial DDT-R allele frequencies, some at Hardy-Weinberg equilibrium and some not. This was to ensure that starting frequencies covered multiple initial conditions, enabling us to critically assess theoretical outcomes empirically over a broad range of starting assumptions. Based on the theoretical expectation of 34 % DDT-R at equilibrium in the absence of DDT (Fig. 1), DDT-R frequency should increase in low initial-frequency experimental populations (LF populations: DDT-R starts at 10 %) and decrease in medium (MF: DDT-R starts at 50 %), and high (HF: DDT-R starts at 90 %) initial-frequency populations (all in Hardy-Weinberg), and should also fall in the nHW populations (non-Hardy-Weinberg: DDT-R starts at 50 %).

**Fig. 2:**
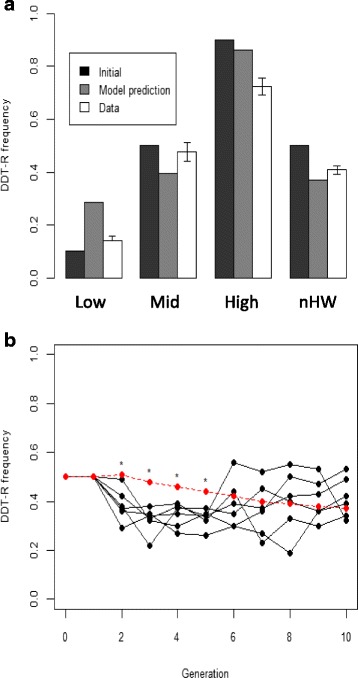
Comparison of model predictions with experimental data for *Canton-S* populations. **(a)** Comparison of final DDT-R frequencies from experimental populations (Low, Mid, High) and nHW, with initial and model prediction frequencies. Empirical data (open bars) is presented as mean frequency with standard error bars. Low, Mid, High population data are for generation 5 while nHW population data are for generation 10. **(b)** Comparison of nHW *Canon-S* population cage allele trajectories with model predictions. Black lines represent DDT-R allele frequencies over 10 generations. All six population cages started at *x*_*R*_ = 0.5 (generation 0) with either 50 RR males and 50 SS females or the reciprocal cross. Hence, all genotypes were RS in generation 1. Red dashed line and square symbols represent the allele frequencies predicted. Population cage frequencies are significantly different from model predictions at generations 2 to 5 (asterisks, t-tests of logit transformed frequency, *p* <0.05) but match model predictions thereafter (all *p* >0.05)

We found that DDT-R frequency increased in seven of eight LF populations, and decreased in MF and HF populations as predicted (Fig. 2a). Additionally, five of the six nHW populations behaved as expected (Fig. 2b). This means that 16 of the 18 populations experienced a shift in allele frequency in the expected direction, qualitatively matching theoretical predictions (one-sided exact binomial test, p <0.001) even though the natural populations are vastly more complicated than our model.

Using *t*-tests of logit-transformed frequency data, there were no significant differences between our observed data and model predictions for the MF (*p* = 0.11), HF (*p* = 0.15) and nHW (*p* = 0.25) populations (Fig. 2a) at the termination of each experiment. However, the final frequencies in the LF populations were significantly lower than predicted (one-sided *t*-test, *p* <0.001). This LF deviation could be due to at least two related factors. First, experimental populations were held at higher density than the original assays used to derive the different fitness parameters [23, 24]. Second, we modelled the relative competitive mating success of DDT-R males based on trials in which two males compete for access to one female [23]. It is far from certain that mating probabilities will be the same at different genotype ratios and densities [30].

It is also important to note that the introgression of DDT-R into our experimental *Canton-S* flies involved a relatively small number of backcross generations, so that a substantial tract (with variable length) of genetic material will be derived from the *Hikone-R* stock and this could account for some of the variability in behaviour in the experiments. Our populations and allele frequency estimation also includes stochastic sampling that is not modelled. Nonetheless, in spite of these differences, there was general agreement between theoretical expectation and experimental data and regardless of whether we start at high or low allele frequency, and whether these are started in Hardy-Weinberg or not, populations tend to behave as predicted.

## Conclusions

We show that sexually antagonistic selection is theoretically able to maintain genetic variation at the *Cyp6g1* locus, and these findings were confirmed in experimental fly populations. To date, only one other study has characterised the evolutionary dynamics of a specific sexually antagonistic allele [31]. That study similarly found that sexual antagonism was able to maintain genetic variation at the antagonistic locus. However, that was an artificial experimentally constructed allele, whereas in our study, we examined the impact of sexually antagonistic selection on a naturally occurring resistance allele.

At present the negative effects of DDT-R on male fitness have only been seen on one of two genetic backgrounds examined (*Canton-S*) [23]. However, as we show here, in principle, sexual antagonism could maintain genetic variation at the locus. Furthermore, and as noted above, the *Canton-S* background has not coevolved with DDT-R, so we can observe the consequences of intralocus conflict before potential modifiers evolve to offset negative fitness effects [25].

Our results have important consequences for the maintenance of genetic variation generally, as intralocus conflict is ubiquitous [7, 9, 13] and conflict resolution is difficult [12, 32]. We have assumed complete dominance of DDT-R (based on its resistance phenotypes), but sex-specific dominance patterns need further investigation as they can have major impact on the genetic architecture of intralocus conflict and may provide an additional avenue through which genetic variation can be maintained [18].

Our findings could also broadly explain the historical DDT-R allele frequency patterns seen in nature and therefore provides the first unifying explanation for a range of somewhat discordant information on DDT-R (the allele was present before DDT use, increases female fitness but did not increase in frequency until widespread DDT use). This has important implications for applied aspects of resistance, including insect pest management, and shows the potential of insect resistance systems to shed light on fundamental questions of evolutionary dynamics. Finally, we show that identifying naturally occurring sexually antagonistic alleles, and estimating selection on them is possible, despite the difficulty associated with mapping sexually antagonistic traits to specific genes [21].

## Methods

### The model

Given the different magnitudes and directions of selection acting at the *Cyp6g1* locus in males and females, it is difficult to predict the invasibility of susceptible populations or how DDT-R frequencies will change in the absence of insecticide selection. Building on a previously published model [33], we modelled the frequency of DDT-R over time in *D. melanogaster* using selection estimates from published fitness determinants documented in the *Canton-S* background in the absence of DDT. Additionally, we considered the effect of including a period of selection with pesticide on allele trajectories, and then by removing DDT selection (as this mimics the current situation), asked if DDT-R could be retained in the absence of this strong source of selection. All aspects of the model were executed using MATLAB [34].

The model terms with default parameter values are outlined in Table 1. Given that there is a competitive mating disadvantage of DDT-R for *Canton-S* males [23], we need to calculate the probability that a mating male has a specific genotype. We do this using the parameter *m,* which represents the mating success of R males relative to S males. The proportion of fathers who carry each genotype is given by the following equations,

3 $$ \begin{array}{l}{y}_{RR}=\frac{m{x}_{RR}}{m\left({x}_{RR}+{x}_{RS}\right)+{x}_{SS}}\\ {}{y}_{RS}=\frac{m{x}_{RS}}{m\left({x}_{RR}+{x}_{RS}\right)+{x}_{SS}}\\ {}{y}_{SS}=1-\left({y}_{RR}+{y}_{RS}\right)\end{array} $$

where *R* represents the DDT-R allele and *S* the susceptible allele. Here we assumed that heterozygote males (*RS*) experience the same mating disadvantage (*m*) as homozygous DDT-R males (*RR*). This assumption is based on the dominant nature of the DDT-R allele with respect to both the resistance [22, 35] and female fitness [24] phenotypes. Male mating probabilities (*y*_*j*_) vary with population genotype frequency (*x*_*i*_) for different values of *m.* For *m* = 1 (i.e. no mating disadvantage), male mating genotype probabilities are equivalent to the genotype frequencies i.e. *y*_*j*_ = *x*_*i*_. Provided there are both resistant and susceptible males in a population, as *m* decreases, the proportion of DDT-R fathers (*y*_*RR*_ and *y*_*RS*_) will be biased downwards (*y*_*RR*_ < *x*_*RR*_ and *y*_*RS*_ < *x*_*RS*_) and the proportion of DDT susceptible fathers (*y*_*SS*_) biased upward (*y*_*SS*_ > *x*_*SS*_).

Now we can calculate the relative mating frequencies (denoted by *λ*) in our population using the DDT-R genotype frequency and male mating probabilities as follows,

4 $$ {\lambda}_{ij}={x}_i{y}_j $$

where the mating frequency subscripts are listed in the order female genotype, male genotype.

Next, DDT-R fitness effects (Additional file 1: Table S1) need to be incorporated into the model in order to predict the genotypic frequencies from one generation to the next. The relative numbers of each genotype eclosing in the next generation can then be calculated, taking into account the mating probabilities and fitness consequences as follows,

5 $$ \begin{array}{l}{n}_{RR}=FP\left({\lambda}_{RRRR}+\raisebox{1ex}{$1$}\!\left/ \!\raisebox{-1ex}{$2$}\right.{\lambda}_{RRRS}+\raisebox{1ex}{$1$}\!\left/ \!\raisebox{-1ex}{$2$}\right.{\lambda}_{RSRR}+\raisebox{1ex}{$1$}\!\left/ \!\raisebox{-1ex}{$4$}\right.{\lambda}_{RSRS}\right)\\ {}{n}_{RS}=FP\left({\lambda}_{RRSS}+\raisebox{1ex}{$1$}\!\left/ \!\raisebox{-1ex}{$2$}\right.{\lambda}_{RRRS}+\raisebox{1ex}{$1$}\!\left/ \!\raisebox{-1ex}{$2$}\right.{\lambda}_{RSRR}+\raisebox{1ex}{$1$}\!\left/ \!\raisebox{-1ex}{$2$}\right.{\lambda}_{RSRS}+\raisebox{1ex}{$1$}\!\left/ \!\raisebox{-1ex}{$2$}\right.{\lambda}_{RSSS}\right)+P\left({\lambda}_{SSRR}+\raisebox{1ex}{$1$}\!\left/ \!\raisebox{-1ex}{$2$}\right.{\lambda}_{SSRS}\right)\\ {}{n}_{SS}=F\left(\raisebox{1ex}{$1$}\!\left/ \!\raisebox{-1ex}{$4$}\right.{\lambda}_{RSRS}+\raisebox{1ex}{$1$}\!\left/ \!\raisebox{-1ex}{$2$}\right.{\lambda}_{RSSS}\right)+\raisebox{1ex}{$1$}\!\left/ \!\raisebox{-1ex}{$2$}\right.{\lambda}_{SSRS}+{\lambda}_{SSSS}\end{array} $$

where *F* = *f* × *e* × *l. f* is the relative fecundity of DDT-R females compared to susceptible females; *e* is the relative viability of eggs laid by DDT-R females; *l* is the relative viability of larvae of DDT-R females compared to susceptible females; and *P* is the relative pupal viability of DDT-R flies compared to susceptible flies. Thus, we effectively census the model population at the adult stage, with relative fitness accrued to males and females being a product of maternal and direct contributions of the *Cyp6g1* genotype as shown in Additional file 1: Table S1.

To obtain the frequency of the genotypes in the next generation we use the following recursions (which can be used via numerical simulation to predict genotype and allele frequencies at specific generations),

6 $$ {x}_i^{\prime }=\raisebox{1ex}{${n}_i$}\!\left/ \!\raisebox{-1ex}{${\Sigma}_i{n}_i$}\right. $$

Now we would like to examine the dynamics of the model, beginning with solving for frequency equilibria $\left({\widehat{x}}_{RR},{\widehat{x}}_{RS},{\widehat{x}}_{SS}\right)$ by letting *x*′ = *x* for each genotype*.* Because the three genotype frequencies must necessarily sum to unity, this non-linear system is effectively a two-variable (*x*_*RR*_, *x*_*RS*_) model and is fully described by the first two genotype recursions. If we represent the functions *x*_*RR*_^′^ and *x*_*RS*_^′^ by *g*_*1*_ and *g*_*2*_, respectively, then there are two conditions, namely ${g}_1\left({\widehat{x}}_{RR},{\widehat{x}}_{RS}\right)={\widehat{x}}_{RR}$ and ${g}_2\left({\widehat{x}}_{RR},{\widehat{x}}_{RS}\right)={\widehat{x}}_{RS}$ which must be satisfied simultaneously at any equilibrium. This was done to obtain an analytical equilibrium solution for DDT-R frequency, *x*_*R*_ (see Additional file 1: equation (S1)).

All initial fitness parameter estimates were derived from previously conducted assays [23, 24]. The relative competitive male mating success, *m*, was derived as the number of mating trials won by resistant males divided by the number won by susceptible males. Relative fecundity, *f*, was derived by dividing the egg count of resistant females by that of susceptible females. The relative viability measures (*e, l, P*), were derived by dividing the resistant viability by the susceptible viability.

To simulate a prolonged period of pesticide selection, the model was initially run for 200 generations, starting at low DDT-R frequency (*x*_*RR*_ = 0, *x*_*RS*_ = 0.001) with all parameters set to default. This represents an initially susceptible population into which the DDT-R allele has been introduced at very low frequency and is allowed to go to an internal equilibrium, representing the situation prior to the use of DDT in the 1940s. After this initial phase a period of ‘DDT selection’ was added by introducing a viability advantage, *D* = 5, that is the mortality ratio of susceptible to resistant flies in the presence of DDT. This ratio is conservative compared to the DDT resistance ratios of Daborn et al. [35]. As the DDT resistance phenotype is dominant, this added viability advantage was assigned to both *RR* and *RS* flies. DDT selection was applied for 300 generations after which time *D* was set to zero and the model run until previous internal equilibrium was achieved.

### Empirical tests of the model in replicate experimental evolution populations

Our model gives specific predictions about the speed with which DDT-R alleles can invade a susceptible population and DDT-R frequency equilibria with the parameter settings employed. How well this describes changes in allele frequencies in real populations is uncertain. As a qualitative test of the model we set up replicate fly populations at known initial DDT-R frequencies and propagated them for five non-overlapping generations to examine DDT-R frequency trajectories over time.

*Canton-S* flies were supplied by Bloomington Stock Center in 2011 and were initially homozygous for the ancestral *Cyp6g1* allele (designated *Cyp6g1-M* by Schmidt et al. [36] and referred to as DDT-S herein) as confirmed by PCR [22]. For the purpose of introgression, we followed McCart et al. [24] in using *Hikone-R* flies (supplied by Bloomington Drosophila Stock Center at Indiana University, Indiana USA in 2011), which are homozygous for the most common resistance-associated *Cyp6g1* allele (designated *Cyp6g1-BA* in a previous study [36] and referred to herein as DDT-R) as confirmed using PCR [22].

DDT-R was introduced to the susceptible background by two replicate crosses each of 25 susceptible stock females × 25 *Hikone-R* males and the reciprocal 25 *Hikone-R* females × 25 susceptible stock males. The 50 flies for each replicate cross were placed in a 10 cm × 6 cm glass jar containing *Drosophila* Quick Mix Medium (Blades Biological Ltd, Edenbridge, Kent, UK), allowed to mate and oviposit for 72 hours and then moved on to a similarly prepared jar – each replicate was moved on twice to maximise offspring production. Immediately following removal of parental flies the inner surface of each jar was laced with DDT by pipetting 500 μl of 60 μg/ml DDT in acetone solution, and rolling until the acetone had fully evaporated. F1 larvae that survived and developed into adults were then backcrossed with the relevant susceptible stock as above. This backcrossing, combined with DDT selection, was carried out for five generations after which offspring were mated in individual pairs and allowed to lay eggs. The parents were then diagnosed for the presence of DDT-R alleles using PCR [22]. The offspring of homozygous DDT-R crosses were then used to found the corresponding DDT-R populations. All populations (DDT-R and DDT-S) were subsequently maintained in (side 30cm) population cages.

We then established eight low frequency (LF) populations (initial DDT-R allele frequency 10 %), two mid-frequency (MF) populations (initial DDT-R allele frequency 50 %) and two high frequency (HF) populations (initial DDT-R allele frequencies 90 %) as follows. Each population was started with two hundred three- to five-day old virgin flies at an even sex ratio with *Cyp6g1* genotypes at Hardy-Weinberg equilibrium frequencies (e.g. RR:RS:SS was 2:36:162 and 50:100:50 for LF and MF replicates, respectively). Populations were reared in vials (diameter 4.5 cm and height 12 cm) with adult flies left to mate and lay eggs for 72 hours, at which time the adults were removed, to limit larval density, and stored at −20 °C. Larvae were allowed to develop, pupate and eclose, and were collected as virgins for four days after initial eclosions. Eighty flies of each sex (n = 160) from the second and third day of eclosion were then haphazardly selected to act as parental flies for the next generation. Non-parental flies (i.e. offspring that were not members of the selected 160) were frozen. The process was repeated for four more generations, after which the populations were terminated. To determine the frequency of *Cyp6g1* genotypes at the end of this period about 50 individual 5th generation flies were analysed by PCR [3] for the presence/absence of the *Accord* LTR-inserted resistance allele.

We also used previously collected population-cage data [37]. The original aim of this population cage experiment was to determine if DDT-R conferred an overall pleiotropic fitness advantage at the population level. Two sets of population cages were established using either 50 RR 5-generation-backcrossed virgin females or males crossed to 50 SS males or 50 SS virgin females (RR × SS and SS × RR), respectively. For each set, three replicate cages were run for a total of six replicate populations (designated here as nHW populations). Flies were left to mate and lay eggs for 72 hours at which time the adults were removed to limit larval density. Following the emergence of the next generation, adult flies were collected for seven days and then used to found a new cage for the next generation. The populations were maintained in this manner for 10 generations. At each generation 80–120 adult offspring were taken from the transfer population to allow allele frequency estimation using PCR [22].

## Additional file

Additional file 1:
**Supplementary supporting information for Rostant et al.**
