# Supplementary material for: Sexual conflict maintains variation at an insecticide resistance locus
Source: BMC Biol. 2015 Jun 29;13:34. doi: 10.1186/s12915-015-0143-3 (PMC4484701; doi:10.1186/s12915-015-0143-3)
Supplement: Additional file 1: — Supplementary supporting information for Rostant et al. [file 12915_2015_143_MOESM1_ESM.docx]

**Sexual conflict maintains variation at an insecticide resistance allele. Rostant et al.**

**Supplementary Materials**

**Table S1:** **Calculating the contribution of each kind of mated pair to each genotype of adult offspring**. Red font indicates DDT-R fitness effects. Note that pupal viability, *P*, is a function of offspring genotype whereas other fitness effects shown here are derived solely from a maternal effect. The numbers to the right are the proportions of offspring from each cross that have a particular genotype (in columns *RR, RS, SS*).

|  | | | *RR* | *RS* | *SS* |
| --- | --- | --- | --- | --- | --- |
| Cross  ♀ × ♂ | Frequency | DDT-R fitness  effects | *P* | | none |
| *RR* × *RR* | *λ_RRRR_* | *F* = *f*×*e*×*l* | 1 |  |  |
| *RR* × *RS* | *λ_RRRS_* | *F* = *f*×*e*×*l* | ½ | ½ |  |
| *RR* × *SS* | *λ_RRSS_* | *F* = *f*×*e*×*l* |  | 1 |  |
| *RS* × *RR* | *λ_RSRR_* | *F* = *f*×*e*×*l* | ½ | ½ |  |
| *RS* × *RS* | *λ_RSRS_* | *F* = *f*×*e*×*l* | ¼ | ½ | ¼ |
| *RS* × *SS* | *λ_RSSS_* | *F* = *f*×*e*×*l* |  | ½ | ½ |
| *SS* × *RR* | *λ_SSRR_* | none |  | 1 |  |
| *SS* × *RS* | *λ_SSRS_* | none |  | ½ | ½ |
| *SS* × *SS* | *λ_SSSS_* | none |  |  | 1 |

**Supplementary Information on explicit solutions for all internal equilibria in the model.**

Where,

And, (S1)

From these the expected equilibria for the default parameter values (Table 1, main text) that satisfy inequalities (1) and (2) can be calculated (see main text).


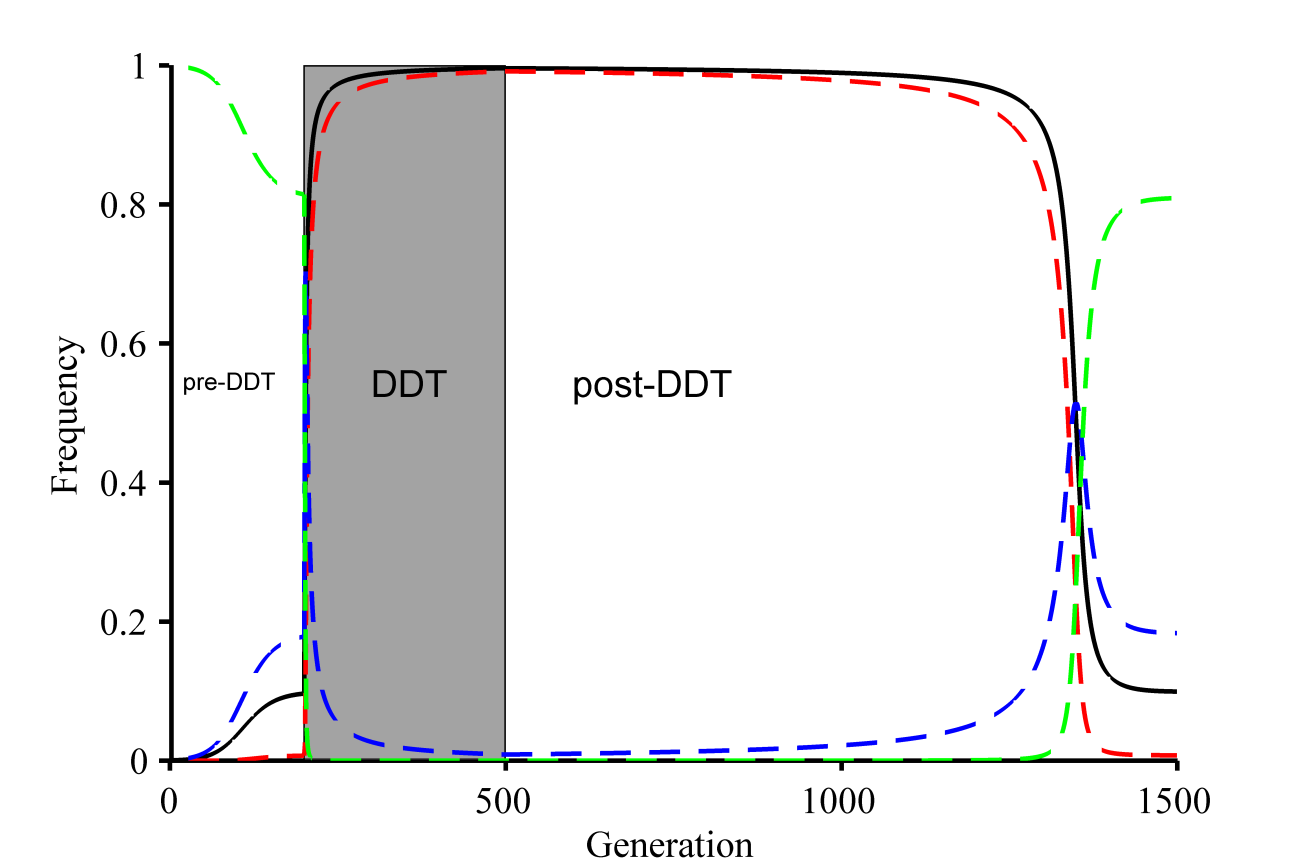


**Figure S1: The effect of DDT selection on DDT-R genotype and allele trajectories in a hypothetical background where the sexually antagonistic effects are conservatively set to be weaker than our empirical data suggest**. In this case hypothetical low equilibrium fitness parameters (*m* = 0.5, *F* = 1.5, *P* = 1.05, *D* = 5) were used, starting from initial genotype frequencies *x_RR_* = 0, *x_RS_* = 0.001, *x_SS_* = 0.999. The red line is the frequency of *x_RR_* , the blue line is *x_RS_* , the green line is *x_SS_* , and the black line is DDT-R. The internal equilibrium of 9.9% in the absence of DDT selection is achieved within the first 200 generations (in the ‘pre-DDT’ period). As with Fig. 1C in the main text, DDT selection (shaded area) starts at generation 201 and ends at generation 500 at which time DDT-R has acquired a frequency of greater than 99%. More than 1000 generations are required ‘post-DDT’ selection for the stable internal equilibrium to be regained (c.f. Fig. 1C, main text).

**Figure S2: McCart et al. (2005) show that Canton-S females have higher fitness when carrying the DDT-R allele, here we show a similar fitness advantage to DDT-R females in another background - the WC genetic background of Smith et al. (2011). Shown here are fitness (fecundity) and viability measures for resistant (RR) and susceptible (SS) females (means ± s.e.).** (a) Female fecundity: Full fecundity data was obtained for 115 mated females, laying for 3 days – this is a good approximation of lifetime offspring production (fitness) (Taylor et al. 2008a,b) and is a proxy for the intrinsic rate of increase of the genotypes (Hunt & Hodgson 2010). Model simplification of a GLM of fecundity against male genotype (RR, SS), female genotype and female size (and all interactions) revealed a significant effect of female resistance genotype on number of eggs laid (F_1,113_ =15.45, p < 0.001) with resistant females (mean eggs laid = 23.92, standard error interval = (22.06, 25.93)) laying more eggs than susceptible females (mean eggs laid = 14.06, standard error interval = (12.56,15.72)). To test if the elevated fecundity of DDT-R females could be eroded by survival costs, we tested egg and larval viability, and found that DDT-R females did not differ from wild-type in these attributes, and hence their fitness would be greater. (b) Egg viability: egg viability data was obtained for 81 females, and simplification of the GLM of egg viability against the same explanatory variables yielded a null minimum adequate model, with no significant effect of female genotype (F_1,79_ = 0.5234, p = 0.47). (c) Combined larval and pupal viability: larvae were collected from 82 females and there was no significant effect of female genotype on combined larval-pupal viability (F_1,80_ = 2.34, p = 0.13). Together these results show that the increased fecundity of DDT-R females is not “lost” through poorer egg or larval viability and hence like DDT-R in *Canton-S*, WC females carrying the DDT-R allele have higher fitness.

References for Supplementary Materials

Hunt, J. & Hodgson, D. J. In *Evolutionary Behavioral Ecology* (eds D.F. Westneat & C.W. Fox) 46-70 (Oxford University Press, 2010).

McCart, C., Buckling, A. & ffrench-Constant, R. H. DDT resistance in flies carries no cost. *Curr. Biol.* **15,** R587-R589 (2005).

Smith, D. T. *et al.* DDT resistance, epistasis and male fitness in flies. *J. Evol. Biol.* **24,** 1351-1362 (2011).

Taylor, M.L. *et al.* Sexual selection and female fitness in *Drosophila simulans. Behav. Ecol. Sociobiol*. 62:721-728 (2008a).

Taylor, M.L. *et al.* Multiple mating increases female fitness in *Drosophila simulans. Anim. Behav*. 76:963-970 (2008b).
